# Quantifying genomic connectedness and prediction accuracy from additive and non-additive gene actions

**DOI:** 10.1186/s12711-018-0415-9

**Published:** 2018-09-17

**Authors:** Mehdi Momen, Gota Morota

**Affiliations:** 1 Department of Animal Science, Faculty of Agriculture, Shahid Bahonar University of Kerman, Kerman, Iran; 2 Department of Animal and Poultry Sciences, Virginia Polytechnic Institute and State University, 175 West Campus Drive, Blacksburg, VA 24061 USA

## Abstract

**Background:**

Genetic connectedness is classically used as an indication of the risk associated with breeding value comparisons across management units because genetic evaluations based on best linear unbiased prediction rely for their success on sufficient linkage among different units. In the whole-genome prediction era, the concept of genetic connectedness can be extended to measure a connectedness level between reference and validation sets. However, little is known regarding (1) the impact of non-additive gene action on genomic connectedness measures and (2) the relationship between the estimated level of connectedness and prediction accuracy in the presence of non-additive genetic variation.

**Results:**

We evaluated the extent to which non-additive kernel relationship matrices increase measures of connectedness and investigated its relationship with prediction accuracy in the cross-validation framework using best linear unbiased prediction and coefficients of determination. Simulated data assuming additive, dominance, and epistatic gene action scenarios and real swine data were analyzed. We found that the joint use of additive and non-additive genomic kernel relationship matrices or non-parametric relationship matrices led to increased capturing of connectedness, up to 25%, and improved prediction accuracies compared to those of baseline additive relationship counterparts in the presence of non-additive gene action.

**Conclusions:**

Our findings showed that connectedness metrics can be extended to incorporate non-additive genetic variation of complex traits. Use of kernel relationship matrices designed to capture non-additive gene action increased measures of connectedness and improved whole-genome prediction accuracy, further broadening the scope of genomic connectedness studies.

**Electronic supplementary material:**

The online version of this article (10.1186/s12711-018-0415-9) contains supplementary material, which is available to authorized users.

Genetic connectedness is used to evaluate the extent to which reliable comparisons of estimated breeding values can be safely performed across management units. The strength of genetic links or connectedness relies on the relatedness of individuals across management units [1]. In turn, genetic evaluations of managed populations such as livestock species rely for their success on sufficient connectedness between different units. In such cases, best linear unbiased prediction (BLUP) provides fair ranking of the estimated breeding values of individuals while minimizing the risk of potential uncertainty in estimated breeding value comparisons [2–4]. The majority of previous studies on connectedness were performed with regard to pedigree relatedness; however, Yu et al. [5] rekindled an interest in this area by evaluating the utility of genome-based connectedness. Using real mice and cattle data, they reported that genomic relatedness enables the enhancement of genetic connectedness measures across management units compared to those obtained from pedigree relationships. This is mainly because genomic information captures relatedness between units that appears disconnected according to the pedigree. The utility of genomic connectedness was further investigated by assessing whether the enhanced estimates of connectedness delivered by genomics also led to an increased accuracy of breeding value prediction [6]. It was found that the use of genomic relatedness yields increased measures of connectedness and improved prediction accuracies (PA) compared to those of pedigree-based models under a purely additive gene action mode when a sufficient number of single-nucleotide polymorphisms (SNPs) is present. This parallels the recent recognition of the impact of non-additive genetic variation marked by SNPs e.g. [7, 8]. By properly accounting for non-additive genetic variation, it is potentially possible to enhance (1) the accuracy of total genetic value prediction, (2) the accuracy of breeding value prediction by clearly separating additive from non-additive genetic variation, and 3) the efficiency of mate allocation procedures as well as crossbreeding or purebred selection schemes [9, 10]. However, the relationship between the estimated level of connectedness and PA in the presence of non-additive genetic variation is less well understood. Accordingly, the objective of the current study was to evaluate the interrelationship between the degree of genomic connectedness and genome-enabled PA by calculating connectedness statistics from either the joint use of additive and non-additive genomic relationship matrices or non-parametric relationship matrices using simulated and real data, further broadening the scope of genomic connectedness studies.

## Methods

### Simulated data

A two-step simulation process was carried out using the QMSim software [11]. A historical population with 1000 individuals was created at the initial generation, followed by a sharp reduction in the population size owing to population bottleneck during generation 1 to 100. This resulted in the population size decreasing to 220 individuals in the last historical generation, creating initial linkage disequilibrium along with mutation and drift. The recent population was formed by randomly sampling 200 females and 10 males from the last historical generation. The individuals were mated for the subsequent five generations with equal probability of males and females, producing a total of 2000 individuals with a structured pedigree for analysis.

The simulated genome consisted of 29 pairs of autosomes each 100 cM long. To mimic a commercial Bovine 54K SNP chip, 1885 bi-allelic SNPs were equally distributed across each chromosome and each chromosome was assigned 65 quantitative trait loci (QTL). Phenotypes were simulated under three different gene action scenarios: (1) additive and dominance (AD), (2) additive, dominance, and epistasis (ADE), and (3) purely epistasis (PE). The simplest quantitative genetic model with main effects (additive and dominance) and epistasis constitutes a two-allele two-locus model. Epistasis was simulated only between pairs of QTL including second order additive $\times$ dominance (A$\times$D) interactions. Five QTL from the 65 on each chromosome (total of 145) were selected to create 10,440 epistatic two-order interactions (145(145−1)/2 = 10,440). The total effect of QTL pairs influencing a given trait was calculated as the sum of all effects using the following model:

$$\begin{aligned} y_i= \sum \limits _{k=1}^{nQTL}{\mathbf {W}}_{\mathbf {a}}{_{ik}}a_{k} + \sum \limits _{k=1}^{nQTL}{\mathbf {W}}_{\mathbf {d}}{_{ik}}d_{k} +\sum \limits _{k=1}^{nQTL}\sum \limits _{k^{\prime }=2}^{nQTL} {\mathbf {l}}_{k}{\mathbf {l}}_{k^{\prime }} ad +\epsilon _{i}. \end{aligned}$$

Here, *a*, *d*, and *ad* are the additive, dominance, and epistatic effects, respectively; ${\mathbf {W}}_{\mathbf {a}}$, ${\mathbf {W}}_{\mathbf {d}}$, and ${\mathbf {l}}_{k}{\mathbf {l}}_{k^{\prime }}$ are SNP codes for additive, dominance, and epistasis, respectively; *k* denotes the *k*th QTL; and *nQTL* is the number of QTL (for the epistatic term, this is only summed over the epistatic QTL). The phenotypic value of each individual $y_i$ was created by adding a normally distributed residual $\epsilon _i \sim N(0,\sigma ^2_{\epsilon })$ to the sum of genetic values. Additive effects were drawn from a Gamma distribution with shape and scale parameters equal to 0.42 and 8.282, respectively [12]. Their effect signs were sampled to be positive or negative with probability 0.5. The dominance effect for the *k*th QTL was determined as the product of the absolute value of the additive QTL effect and the degree of dominance $d_{k}=\delta _k\mid a_{k}\mid$ [13, 14]. Here, $\delta _k$ is the degree of dominance sampled from a normal distribution with $\delta _k \sim N(0,1)$. The epistatic effects were drawn from a normal distribution with $N(0.02,\sigma ^2=0.03)$ [14]. Additive and dominance components were simulated for the AD scenario; additive, dominance, and epistatic components were included for the ADE scenario; and only epistasis was considered for the PE scenario. Two broad-sense heritability levels ($H^2$) equal to 0.4 and 0.8 were simulated, with the partitioning of variance components shown in Table 1. We considered phenotypic variance equal to unity and simulated genetic variance according to the proportion of phenotypic variance explained by additive, dominance, and epistatic QTL effects: $\sigma ^2_a = \sum _k 2p_kq_k\alpha ^2_k$, $\sigma ^2_d = \sum _k [2p_kq_kd_k]^2$, and $\sigma ^2_{ad} = 2\sum _k \sum _{k^{\prime }} p^2_k p_{k^{\prime }} q_{k^{\prime }}(\alpha _k d_{k^{\prime }})^2$, where $\alpha =[a + d(q-p)]^2$ is the allele substitution effect, and *p* and *q* are minor and major allele frequencies, respectively [15, 16].

**Table 1:** Simulated heritability value for each gene action scenario

$H^2$	Gene action	$h^2_A$	$h^2_D$	$h^2_E$
0.4	AD	0.3	0.1	-
	ADE	0.2	0.1	0.1
	PE	–	–	0.4
0.8	AD	0.6	0.2	-
	ADE	0.4	0.2	0.2
	PE	–	–	0.8

$H^2$, $h^2_A$, $h^2_D$, and $h^2_E$ are broad-sense, additive, dominance, and epistatic heritabilities, respectively. Gene action scenarios AD, ADE, and PE denote additive and dominance, additive, dominance, and epistasis, and purely epistasis, respectively

### Real data

For real data analysis, publicly available PIC swine data was used [17]. We analyzed five traits, T1, T2, T3, T4, and T5, with the corresponding number of individuals equal to 2804, 2715, 3141, 3184, and 3184. Their heritability values were 0.03, 0.23, 0.20, 0.32, and 0.36, respectively. It has been shown that this dataset exhibits a small to moderate amount of dominance genomic variation [18, 19]. Therefore, this dataset was considered suitable to test the extent to which the use of a non-additive genomic kernel relationship matrix might increase the capturing of connectedness measures. After removing SNPs with a minor allele frequency lower than 0.05, 52,842 SNPs remained for the analysis.

### Management unit simulation

**Fig. 1:**
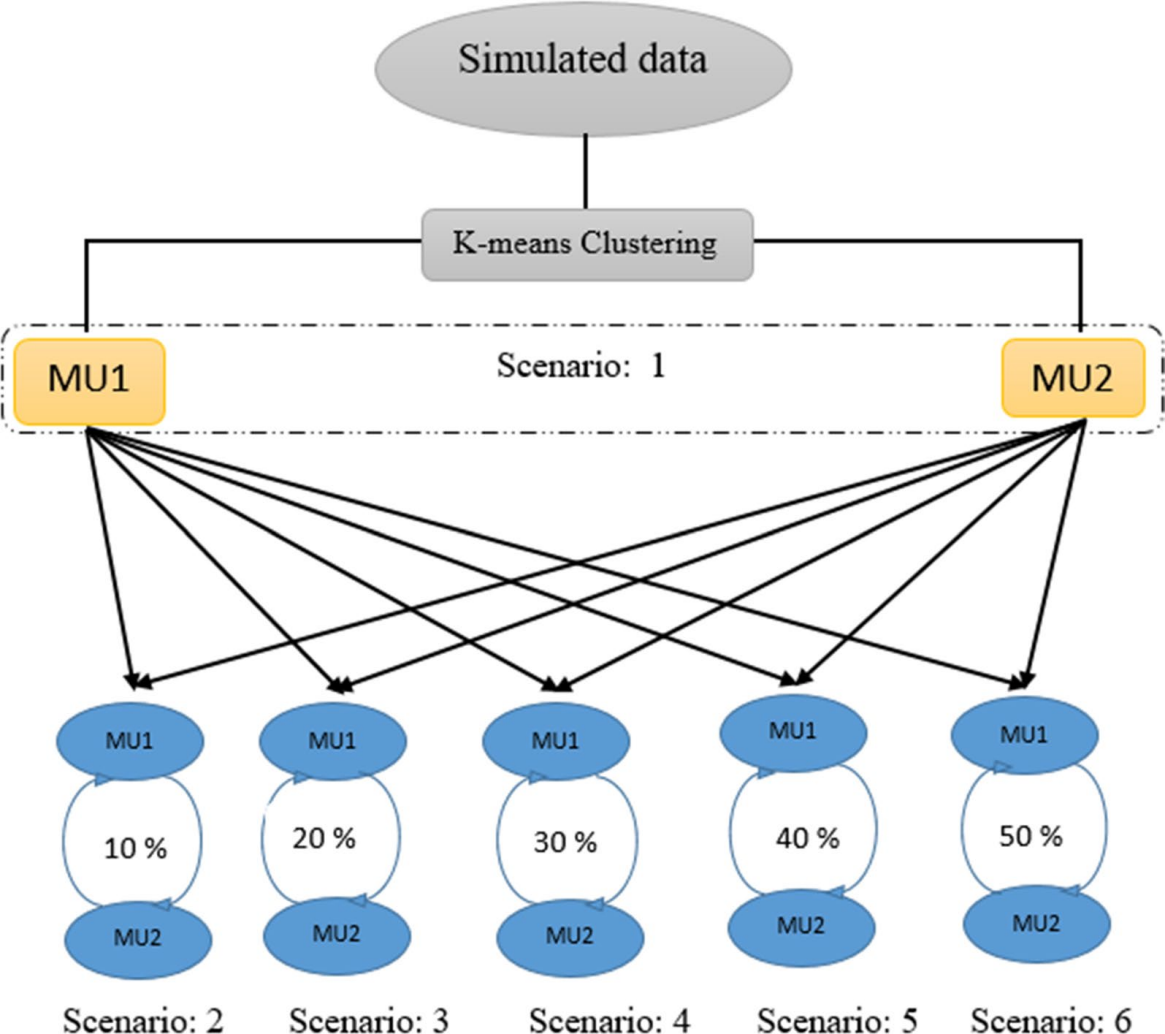
Simulated management units (MU). Scenario 1: Disconnected management units MU1 and MU2. Scenario 2: 10% of individuals were exchanged between MU1 and MU2. Scenario 3: 20% of individuals were exchanged between MU1 and MU2. Scenario 4: 30% of individuals were exchanged between MU1 and MU2. Scenario 5: 40% of individuals were exchanged between MU1 and MU2. Scenario 6: 50% of individuals were exchanged between MU1 and MU2

The management units were simulated according to the approach in Yu et al. [5] for simulated and real data. We clustered all individuals into management unit 1 (MU1) and management unit 2 (MU2) using the *K*-means clustering algorithm applied to a numerator relationship matrix computed from pedigree data such that the overall level of relatedness between individuals in different management units is minimized. There was no exchange of individuals between MU1 and MU2 in scenario 1 (S1), which served as a least connected design. An additional five management unit scenarios (S2 to S6) were considered by exchanging 10, 20, 30, 40, and 50% of individuals between MU1 and MU2 as shown in Fig. 1.

### Genomic relationship kernel matrix

Three types of genomic relationship kernel matrices (${\mathbf {K}}$) were used in the present study.

The additive genomic relationship matrix (${\mathbf {K}} ={\mathbf {G}}$) was used to capture the pattern of additive inheritance ${\mathbf {G}} = {\mathbf {W}}_{\mathbf {a}}{\mathbf {W}}_{\mathbf {a}}^{\prime } / 2 \sum _{k=1}^{m} p_k(1-p_k)$, where ${\mathbf {W}}_{\mathbf {a}}$ is the centered marker incidence matrix taking values of $0-2p_k$ for zero copies of the reference allele, $1-2p_k$ for one copy of the reference allele, and $2-2p_k$ for two copies of the reference allele [20]. Here, $p_k$ is the allele frequency at SNP $k = 1, \ldots , m$. The dominance genomic relationship matrix (${\mathbf {K}} = {\mathbf {D}}$) aimed at capturing dominance gene action ${\mathbf {D}}={\mathbf {W}}_{\mathbf {d}}{\mathbf {W}}_{\mathbf {d}}^{\prime } / \sum _{k=1}^{m}(2p_k(1-p_k))^2$, where ${\mathbf {W}}_{\mathbf {d}}$ is the dominance marker incidence matrix defined according to Vitezica et al. [21]. The additive by dominance genomic relationship matrix was constructed as ${\mathbf {G}}\#{\mathbf {D}}$, where $\#$ denotes the Hadamard product [22].

#### Gaussian kernel

The Gaussian kernel (${\mathbf {K}} = {\mathbf {GK}}$) is equivalent to modeling epistatic gene action up to an infinite order by taking the Hadamard product between ${\mathbf {G}}$ matrices when SNPs were coded in an additive manner [23]. It is also known as a space continuous version of the diffusion kernel, which is deployed on graphs [24]. The Gaussian kernel between a pair of individuals *i* and *j* with their genotype vectors ${\mathbf {w}}_i \in (0,1,2)$ and ${\mathbf {w}}_j \in (0,1,2)$ is given by:

$$\begin{aligned} {\mathbf {GK}}({\mathbf {w}}_{i}, {\mathbf {w}}_{j})&= \exp (- \theta d_{ij}^2) \\&= \prod _{k=1}^{m} \exp (-\theta (w_{ik} - w_{jk})^2), \end{aligned}$$

where $d_{ij} = \sqrt{ (w_{i1} - w_{j1})^2 + \cdots + (w_{ik} - w_{jk})^2 + \cdots + (w_{im} - w_{jm})^2}$ is the Euclidean distance and $\theta$ is the smoothing parameter. Large $\theta$ leads to **GK** entries closer to 0 (i.e., local kernel) and smaller $\theta$ produces entries closer to 1 (i.e., global kernel). Therefore, $\theta$ controls the extent of genomic similarity between individuals.

### Coefficient of determination

Consider a standard BLUP model, ${\mathbf {y}}={\mathbf {Xb}} + {\mathbf {Zu}} + \varvec{\epsilon }$, where ${\mathbf {y}}$ is the vector of the phenotypes, ${\mathbf {X}}$ and ${\mathbf {Z}}$ are the incidence matrices for systematic and random effects, respectively, **b** and **u** are the vectors of systematic effects and genetic values, and $\varvec{\epsilon }$ is the vector of residuals. By defining $\text {var}({\mathbf {u}}) = {\mathbf {K}}\sigma ^2_u$ we have:

$$\begin{aligned} \text {BLUP}(u)&= \sigma ^2_u{\mathbf {K}}{\mathbf {Z^{\prime }}}{\mathbf {V}}^{-1}({\mathbf {y}}-{\mathbf {X}}{\hat{\mathbf {b}}}) \\&= \sigma ^2_u{\mathbf {K}}{\mathbf {Z}}^{\prime }{\mathbf {Py}} \\ \text {var}({\hat{\mathbf {u}}})&= \sigma ^2_u{\mathbf {K}} {\mathbf {Z}}^{\prime }{\mathbf {PZ}} {\mathbf {K}}\sigma ^2_u \end{aligned}$$

where $\sigma ^2_u$ is the variance associated with a kernel matrix ${\mathbf {K}}$, ${\mathbf {V}}$ is the variance of ${\mathbf {y}}$, and ${\mathbf {P}}={\mathbf {V}}^{-1}-{\mathbf {V}}^{-1}{\mathbf {X}}({\mathbf {X}}^{\prime }{\mathbf {V}}^{-1}{\mathbf {X}})^{-}{\mathbf {X}}^{\prime }{\mathbf {V}}^{-1}$ [25]. Recall that since $\text {cov}({\hat{\mathbf {u}},\mathbf {u}^{\prime }})$ = $\text {cov}({\mathbf {u}}^{\prime },{\hat{\mathbf {u}}})$ = $\text {var}({\hat{\mathbf {u}}})$, the prediction error variance (PEV) of ${\mathbf {u}}$ is given by:

$$\begin{aligned} \text {PEV}&= \text {var}({\hat{\mathbf {u}}-{\mathbf {u}}}) \\&= \text {var}({\hat{\mathbf {u}}}) + \text {var}({\mathbf {u}}) - 2\text {cov}({\hat{\mathbf {u}},{\mathbf {u}}^{\prime }}) \\&= \text {var}({\mathbf {u}}) - \text {var}({\hat{\mathbf {u}}}) \\&={\mathbf {K}}\sigma ^2_u -\sigma _u^2{\mathbf {KZ}}^{\prime }{\mathbf {PZK}}\sigma _u^2, \end{aligned}$$

where $\mathbf K$ can be any positive (semi)definite relationship matrix between pairs of individuals discussed earlier.

The generalized coefficient of determination (CD), which is also known as the square of the correlation between the predicted and the true difference in the genetic values, was used to quantify connectedness. CD of the contrast between management units *l* and $l^{\prime }$ consisting of $n_l$ and $n_{l^{\prime }}$ individuals is given by [26, 27]:

$$\begin{aligned} \text {CD}&= \frac{{\mathbf {x}}_{ll^{\prime }} \text {var}(\hat{{\mathbf {u}}}){\mathbf {x}}_{ll^{\prime }}}{{\mathbf {x}}_{ll^{\prime }}\text {var}({\mathbf {u}}){\mathbf {x}}_{ll^{\prime }}} \\&=\frac{{\mathbf {x}}_{ll^{\prime }}[\text {var}({\mathbf {u}})-\text {var}(\hat{{\mathbf {u}}}-{\mathbf {u}})]{\mathbf {x}}_{ll^{\prime }}}{{\mathbf {x}}_{ll^{\prime }}\text {var}({\mathbf {u}}){\mathbf {x}}_{ll^{\prime }}} \\&= 1 - \frac{{\mathbf {x}}_{ll^{\prime }}[\text {var}(\hat{{\mathbf {u}}}-{\mathbf {u}})]{\mathbf {x}}_{ll^{\prime }}}{{\mathbf {x}}_{ll^{\prime }}\text {var}({\mathbf {u}}){\mathbf {x}}_{ll^{\prime }}}, \end{aligned}$$

where ${\mathbf {x}}$ is the contrast vector involving $1/n_{l}$, $-1/n_{l^{\prime }}$ and 0 corresponding to individuals belonging to *l*th, $l^{\prime }$th, and the remaining units. Here, the sum of contrast vector elements is zero. The greater the CD of contrast, the greater the connectedness. A large CD is expected when prediction error covariance in the numerator is large, reflecting errors that are in the same direction between units. Alternatively, the measure of CD decreases when the relationship between individuals across units is large in the denominator. Therefore, the CD of contrast combines the prediction error variance of the difference (PEVD) [2] and genetic variability. This metric was chosen because it was found to represent the most stable connectedness metric in a recent study [5].

### Connectedness measures and prediction accuracy

Measures of CD between MU1 and MU2 were inferred from estimated variance components followed by assessing genomic PA by two-fold cross-validation using a BLUP type model. In the first fold, MU1 was treated as a training set and MU2 was treated as a testing set. This was reversed in the second fold such that MU2 was used to train the model and MU1 was used to test prediction performance. The multi-kernel ${\mathbf {G}}$ and ${\mathbf {D}}$ approach in the AD scenario, the multi-kernel ${\mathbf {G}}$, ${\mathbf {D}}$, and ${\mathbf {G}}\#{\mathbf {D}}$ approach in the ADE scenario, and the ${\mathbf {GK}}$ matrix in the PE scenario were benchmarked against the baseline ${\mathbf {G}}$ matrix (i.e., genomic BLUP). Note that the use of ${\mathbf {GK}}$ corresponds to fitting a reproducing kernel Hilbert spaces regression (e.g. [28]). For a multi-kernel approach, we weighted each kernel by its relative contribution to the marked total genetic variation, also known as kernel averaging or multiple kernel learning [29], to measure connectedness and assess PA. For instance, the kernel matrix ${\mathbf {K}} = \frac{\sigma ^2_g}{\sigma ^2_g + \sigma ^2_d} {\mathbf {G}} + \frac{\sigma ^2_d}{\sigma ^2_g + \sigma ^2_d} {\mathbf {D}}$ was used when ${\mathbf {G}}$ and ${\mathbf {D}}$ were fitted together, where $\sigma ^2_g$ and $\sigma ^2_d$ were additive and dominance genomic variances, respectively. PA was obtained as the correlation between true and predicted genetic values for the simulated data averaged across 10 replicates (cor(${\mathbf {g}}, \hat{{\mathbf {g}}}$)) and the correlation between phenotypes and predicted genetic values for the real data (cor(${\mathbf {y}}, \hat{{\mathbf {g}}}$)).

## Results

### AD scenario

**Fig. 2:**
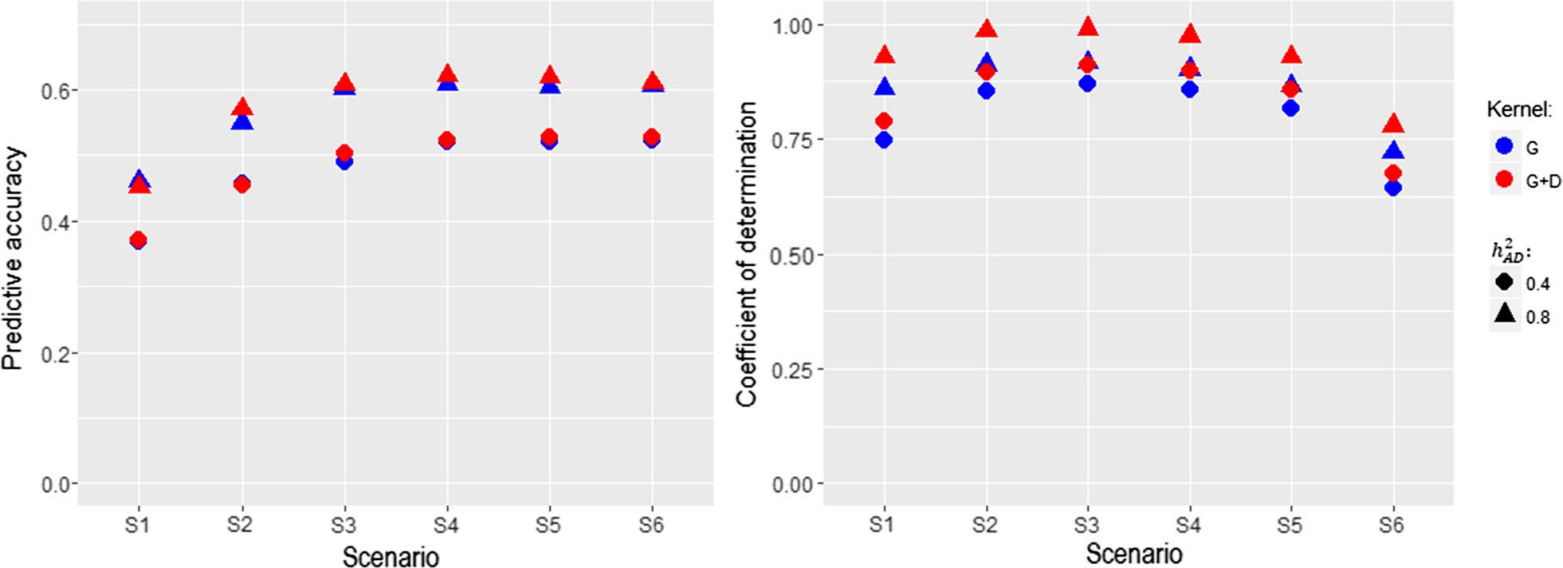
Relationship between prediction accuracies (left panel) and connectedness measures (right panel) under an additive and dominance scenario. The magnitude of the relationship level was steadily increased from scenario 1 (S1) to scenario 6 (S6). ${\mathbf {G}}$: additive genomic kernel relationship matrix. ${\mathbf {D}}$: dominance genomic kernel relationship matrix. $h^2_{AD}$: broad-sense heritability including additive and dominance variation

The relationships between CD and PA across the six management unit simulation scenarios (S1 to S6) are shown in Fig. 2. The joint fit of ${\mathbf {G}}$ and ${\mathbf {D}}$ kernel relationship matrices was benchmarked using the ${\mathbf {G}}$ matrix alone. A sharp increase in PA was observed with the increasing proportion of exchanged individuals from S1 to S3, which reached a plateau after 30% exchange rate between MU1 and MU2 in S4. Overall, PA improved as more individuals between MU1 and MU2 were shared. Higher PA values were achieved by accounting for dominance ${\mathbf {G}}+{\mathbf {D}}$ compared to ${\mathbf {G}}$ alone for the two heritability levels considered (0.4 and 0.80). The lowest PA (0.368) was obtained in S1 with ${\mathbf {G}}$ and the highest PA (0.632) was obtained in S4 with ${\mathbf {G}}+{\mathbf {D}}$.

For the measures of connectedness, there was a good agreement between increasing the rate of exchange and stronger measures of connectedness up to S3. However, the estimates of CD increased up to scenario S3, followed by a decrease from scenario S4 onward because CD penalizes connectedness measures when two units are genetically close. The results showed that establishing genetic links between management units by exchanging more individuals created more genetic similarity on one side and reduced genetic variability on the other side, resulting in lower CD values. CD of contrast measured by ${\mathbf {G}}+{\mathbf {D}}$ captured stronger connectedness than that of ${\mathbf {G}}$ consistently across all scenarios (S1 to S6). The largest measured CD (0.989) was obtained with ${\mathbf {G}}+{\mathbf {D}}$ in S3, and the smallest CD (0.64) was obtained with ${\mathbf {G}}$ in S6. Overall, accounting for dominance variation increased PA and measures of CD. The relationship between PA and CD was positively associated up to S3; then, whereas PA continued to increase, CD began to level off.

### ADE scenario

**Fig. 3:**
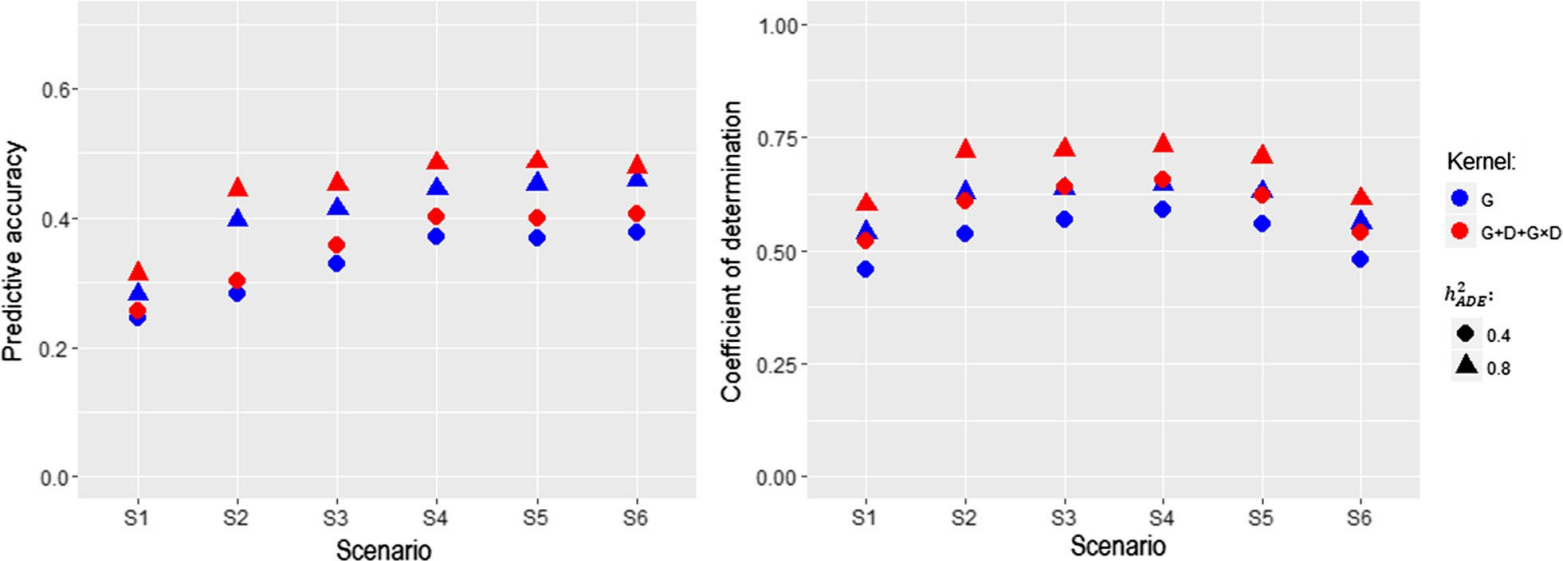
Relationship between prediction accuracies (left panel) and connectedness measures (right panel) under an additive, dominance, and epistasis scenario. The magnitude of the relationship level was steadily increased from scenario 1 (S1) to scenario 6 (S6). ${\mathbf {G}}$: additive genomic kernel relationship matrix. ${\mathbf {D}}$: dominance genomic kernel relationship matrix. ${\mathbf {G}} \times {\mathbf {D}}$: additive $\times$ dominance genomic kernel relationship matrix. $h^2_{ADE}$: broad-sense heritability including additive, dominance, and epistatic variation

The results of PA and CD from the ADE scenario are shown in Fig. 3. We found that the overall pattern resembled that of the AD scenario. That is, with increasing degree of similarity among management units, PA increased and then reached a plateau after S4. The highest PA (0.731) was obtained with ${\mathbf {G}}+{\mathbf {D}}+{\mathbf {G}}\#{\mathbf {D}}$ kernel matrices in S4 and the smallest PA (0.245) with ${\mathbf {G}}$ in S1. The PA results suggested that increasing the number of linking individuals improves PA and the use of non-additive genomic relationship matrices simultaneously further increased PA.

In comparison, measures of CD were strengthened with the increase of linking individuals up to S4, followed a decreasing tendency, similar to the pattern observed in the AD scenario. Improved capture of connectedness was achieved by explicitly accounting for additive, dominance, and epistasis variations compared to additive only. The greatest and weakest measures of connectedness were observed with ${\mathbf {G}}+{\mathbf {D}}+{\mathbf {G}}\#{\mathbf {D}}$ (0.731) in S4 and with ${\mathbf {G}}$ (0.456) in S1, respectively.

### PE scenario

**Fig. 4:**
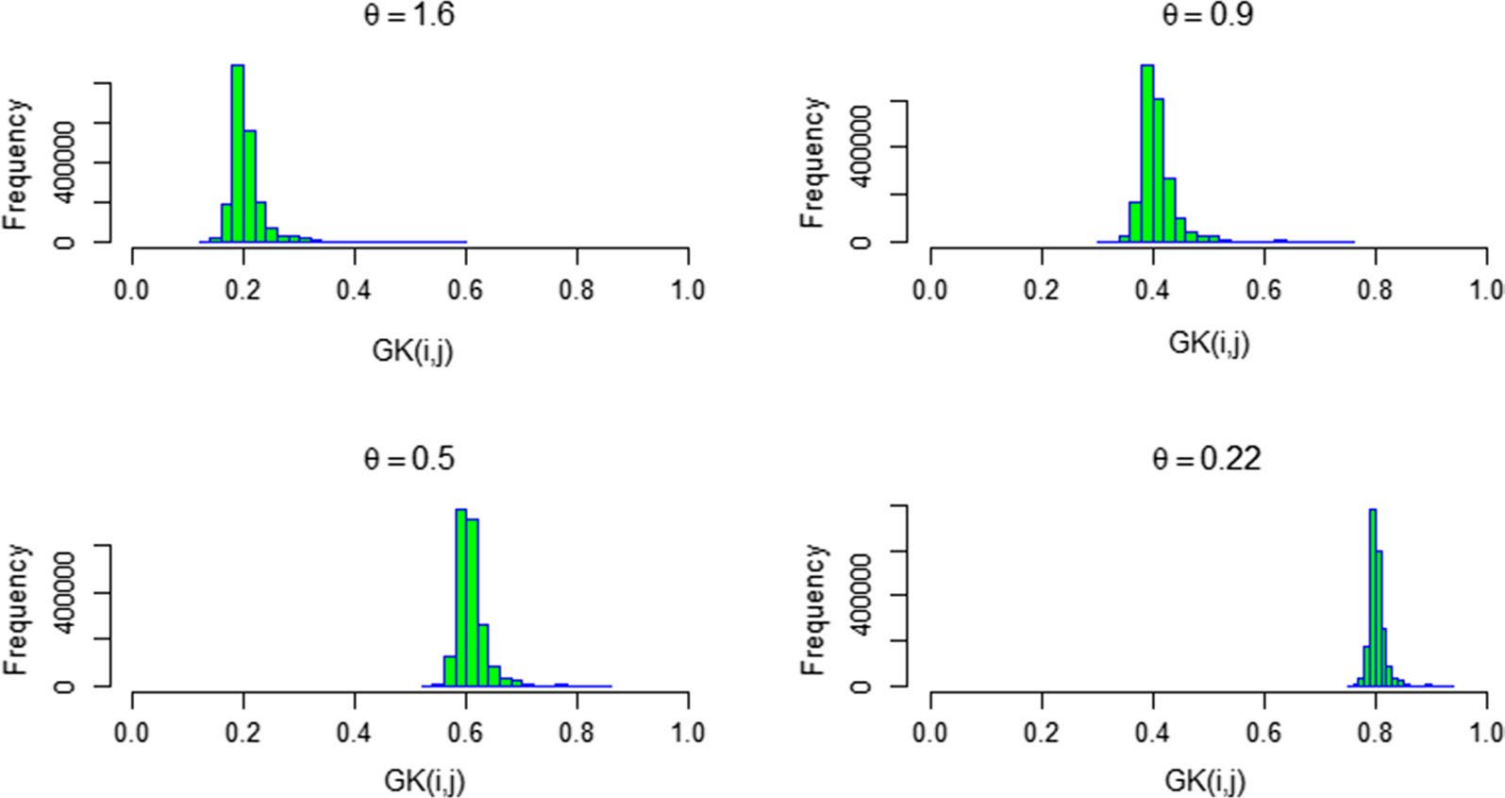
Histogram of off-diagonal elements between individual *i* and *j* for the Gaussian kernel matrix ${\mathbf {GK}}$(i, j) with different smoothness parameters $\theta$ = 1.6, 0.9, 0.5, and 0.22

**Fig. 5:**
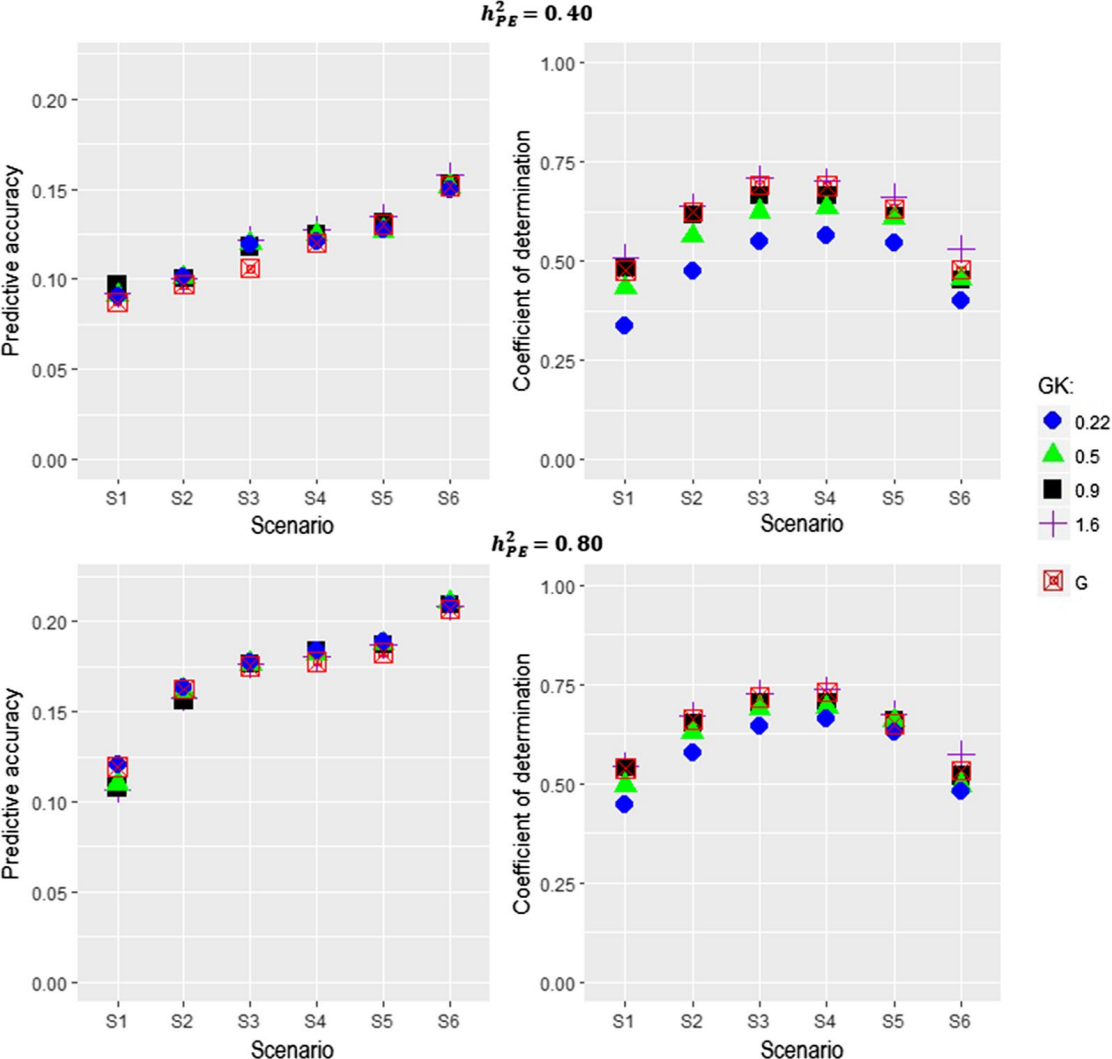
Relationship between prediction accuracies (left panel) and connectedness measures (right panel) under a purely epistasis scenario. The magnitude of the relationship level was steadily increased from scenario 1 (S1) to scenario 6 (S6). ${\mathbf {GK}}$: Gaussian kernel relationship matrix with the smoothness parameters $\theta$ = 1.6, 0.9, 0.5, and 0.22. ${\mathbf {G}}$: additive genomic kernel relationship matrix. $h^2_{PE}$: broad-sense heritability including epistatic variation

Performance of ${\mathbf {GK}}$ and ${\mathbf {G}}$ was compared in the PE scenario. We considered different values for the smoothness parameter $\theta$ ranging from 0.22, 0.5, and 0.9 to 1.6. These $\theta$ values were chosen such that the averages of off-diagonal elements corresponded to 0.8, 0.6, 0.4, and 0.2 covering global to local kernels (Fig. 4). The relationship between PA and CD for ${\mathbf {GK}}$ and ${\mathbf {G}}$ is shown in Fig. 5. For $H^2=0.4$, the results from the PE scenario were similar to those of AD and ADE scenarios, showing a higher PA with an increasing number of linking individuals. A $\theta$ equal to 1.6 produced the highest overall PA. Altogether, these results demonstrate the usefulness of ${\mathbf {GK}}$ to capture information arising from non-additive genetic variation. The advantage of ${\mathbf {GK}}$ over ${\mathbf {G}}$ for PA was less obvious when heritability was high ($H^2=0.8$).

The right side panel in Fig. 5 illustrates how $\theta$ impacts the measures of connectedness under the PE scenario. For $H^2 =0.40$, the largest CD value was obtained in S3 with ${\mathbf {GK}}(\theta ) = 1.6$, and the smallest values were observed in S1 and S6 with ${\mathbf {GK}}(\theta ) = 0.22$. The connectedness measures from ${\mathbf {G}}$ were between these two extreme ${\mathbf {GK}}$. Again, the highest PA was observed in S6 whereas the highest CD was observed in S3. This is because CD accounts for the reduction of connectedness owing to low genetic diversity [27]. A similar pattern was observed for $H^2=0.8$, highlighting that the utility of ${\mathbf {GK}}$ to capture connectedness under non-additive gene actions also holds for a highly heritable trait.

### Real data

**Fig. 6:**
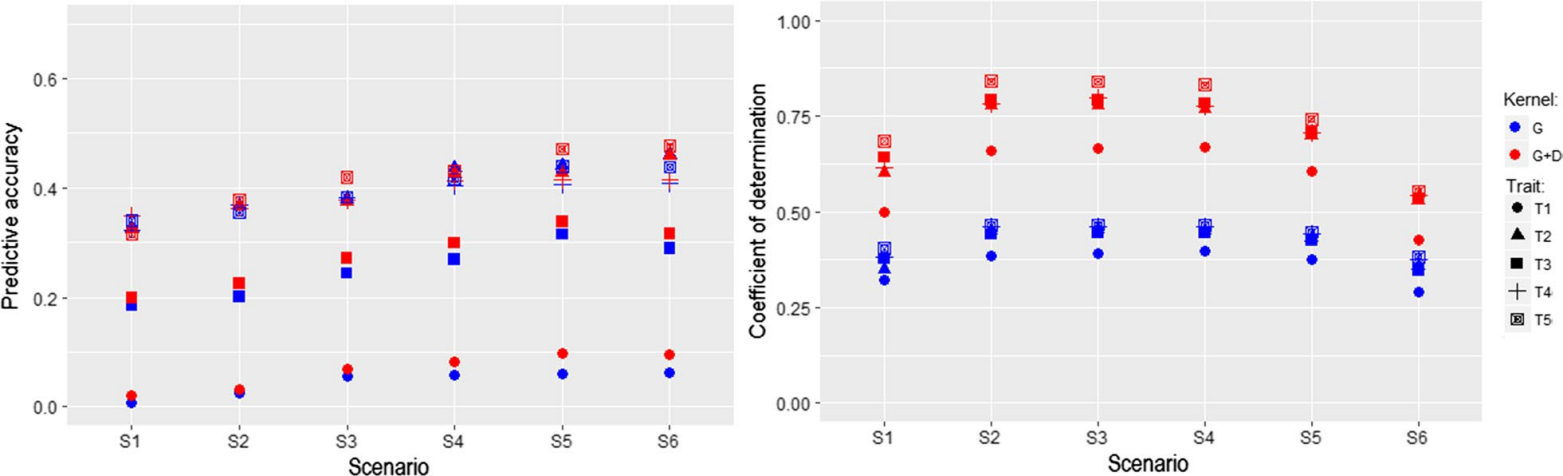
Relationship between prediction accuracies (left panel) and connectedness measures (right panel) in the real swine data. The magnitude of the relationship level was steadily increased from scenario 1 (S1) to scenario 6 (S6). ${\mathbf {G}}$: additive genomic kernel relationship matrix. ${\mathbf {D}}$: dominance genomic kernel relationship matrix. T1 to T5 denote five different traits analyzed in this study

The results from real data are shown in Fig. 6. As more individuals between the two units were exchanged, PA increased across all traits until a maximum was reached whereas CD started to drop in S5. Fitting ${\mathbf {G}}$ and ${\mathbf {D}}$ simultaneously yielded better prediction in almost all cases and also captured greater amounts of connectedness than those of ${\mathbf {G}}$ alone. Traits with a higher heritability (e.g. T4 and T5) presented higher PA and greater CD levels than those with a lower heritability (e.g. T1). The results from real data analysis corroborated the utility of the multi-kernel approach from the simulation study.

## Discussion

The assessment of genetic connectedness originated from testing the estimability of linear functions of fixed effects in *n*-way cross classifications to determine the absence or presence of connectedness [30, 31]. It was subsequently extended to the random effects framework [1] to quantify the uncertainty associated with the accuracy of breeding value comparisons involving different management units. In this sense, connectedness is a measure germane to the capability to have estimable comparisons [3]. In the genomics era, the concept of genetic connectedness offers insights on two aspects of the prediction of genetic values. The first is relevant to improving the quality of genomic breeding value comparisons [5, 32] whereas the other is related to improving the accuracy of genomic prediction [33]. Notably, it is possible to reconcile these two items by quantifying a genomic connectedness level between reference and validation sets in the whole-genome prediction paradigm. Toward this end, Yu et al. [6] investigated the relationship between connectedness measures and PA using pedigree and genomic information under an additive model.

Concurrently, it has been shown that whole-genome prediction models designed to capture non-additivity yield slightly to moderately higher PA than additive counterparts when the underlying genetic architecture is governed by dominance or epistasis e.g. [28, 34]. Although the extent of non-additive genetic variance may not be big in general, this type of variance is particularly important for fitness-related traits [35]. These recent findings served as the impetus for the present study, extending the scope of connectedness applications by further considering non-additive genetic variation.

We observed that the inclusion of non-additive genetic relationship kernel matrices or non-parametric relationship matrices in a BLUP type model increased PA as more individuals were exchanged between MU1 and MU2, and that this was associated with stronger measures of connectedness up to S3 or S4. This reinforced the view that the commonly observed higher prediction performance in non-additive or non-parametric models in the presence of non-linear gene action is due to improved capturing of connectedness between units. We also found that the choice of smoothness parameter $\theta$ not only influences PA but also the extent of CD. This indicates the importance of the smoothness parameter in evaluating PA and CD, especially when a complex trait is controlled by non-additive gene actions. In general, our results showed that when the optimum $\theta$ is selected, PA and CD of **GK** will be better than those of **G**, and that even **GK** constructed from additive coding of SNPs only captures additive by additive epistasis theoretically [23]. We note that many studies have shown that PA decreases when the reference population has a lower relatedness to the validation population e.g. [36, 37]. This is equivalent to when two units exhibit weak connectedness. Use of connectedness thereby opens up the possibility for an alternative way to measure the strength of relationship between these two populations instead of using an average relationship.

Moreover, once the rate of exchange reached S3 or S4, the estimated level of CD gradually leveled off in all management unit simulation scenarios, contrary to PA. This is because when there are sufficient numbers of individuals linking MU1 and MU2, the denominator of CD becomes smaller thus increasing the second term, which in turn renders the CD of contrast to become small. This agrees with the findings in other studies dealing with only additive genetic variation [5, 6]. Together, these findings suggest that the use of CD holds great potential to identify an optimal breeding program design in terms of genetic diversity while maximizing PA, whereas other connectedness metrics such as PEVD aim at increasing PA regardless of how closely individuals between units become related [5]. Note that PA is one of the criteria to determine the most appropriate model to fit (for example, ${\mathbf {G}}$ vs. ${\mathbf {G}} + {\mathbf {D}}$). Once the model is chosen, CD can be used to identify an appropriate level of relatedness or diversity between two units while maintaining high PA.

Although we applied *K*-means clustering of a numerator relationship matrix, the choice of ${\mathbf {K}}$ for clustering may impact our results. Thus, we further constructed management units based on clustering of ${\mathbf {G}}$ or ${\mathbf {G}}$ + ${\mathbf {D}}$ under the AD scenario. As shown in Figures S1 and S2 (see Additional file 1: Figures S1 and S2), *K*-means clustering of ${\mathbf {G}}$ or ${\mathbf {G}}$ + ${\mathbf {D}}$ produced patterns of PA and CD that are similar to those generated using the numerator relationship. We also repeated our analyses using forward validation rather than *K*-means clustering. We treated 1200 individuals in generations 1 to 3 as the training set (MU1) and 800 individuals in generations 4 to 5 as the testing set (MU2) under the AD scenario. We found that using ${\mathbf {G}} + {\mathbf {D}}$ yielded higher PA and greater amount of CD compared to using ${\mathbf {G}}$ (Additional file 1: Figure S3).

The utility of genomic connectedness does not preclude its application in management units. For instance, connectedness measured by CD is currently gaining recognition for training population formation in plant breeding [38]. We contend that the use of CD holds promise to tackle a multitude of challenges related to increasing genomic prediction while maintaining genetic diversity.

## Conclusion

Here, the genetic connectedness metric, CD, was used to assess genomic connectedness measures between reference and validation sets in a whole-genome prediction framework using simulated and real data in the presence of non-additive gene action. Joint fitting of additive and non-additive genomic kernel relationship matrices or non-parametric relationship matrices could yield enhanced capture of connectedness and improved PA compared to those obtained through baseline additive models. Our approach shows promise to measure connectedness levels and investigate their relationship with genomic PA when the linear assumption of genotype-phenotype mapping may not hold.

## Additional file

**Additional file 1: Figure S1.** Relationship between prediction accuracies (left panel) and connectedness measures (right panel) under an additive and dominance scenario based on the *K*-means clustering using the genomic relationship matrix. The magnitude of the relationship level was steadily increased from scenario 1 (S1) to scenario 6 (S6). ${\mathbf {G}}$: additive genomic kernel relationship matrix. ${\mathbf {D}}$: dominance genomic kernel relationship matrix. $h^2_{AD}$: broad-sense heritability including additive and dominance variation. **Figure S2.** Relationship between prediction accuracies (left panel) and connectedness measures (right panel) under an additive and dominance scenario based on the *K*-means clustering using the multikernel genomic and dominance relationship matrix. The magnitude of the relationship level was steadily increased from scenario 1 (S1) to scenario 6 (S6). ${\mathbf {G}}$: additive genomic kernel relationship matrix. ${\mathbf {D}}$: dominance genomic kernel relationship matrix. $h^2_{AD}$: broad-sense heritability including additive and dominance variation. **Figure S3.** Relationship between prediction accuracies (left panel) and connectedness measures (right panel) under an additive and dominance scenario based on forward validation. ${\mathbf {G}}$: additive genomic kernel relationship matrix. ${\mathbf {D}}$: dominance genomic kernel relationship matrix. $h^2$: heritability.
