# Supplementary material for: Quantifying genomic connectedness and prediction accuracy from additive and non-additive gene actions
Source: Genet Sel Evol. 2018 Sep 17;50:45. doi: 10.1186/s12711-018-0415-9 (PMC6142710; doi:10.1186/s12711-018-0415-9)
Supplement: Supplementary file 1 — Additional file 1: Figure S1. Relationship between prediction accuracies (left panel) and connectedness measures (right panel) under an additive and dominance scenario based on the K-means clustering using the genomic relationship matrix. The magnitude of the relationship level was steadily increased from scenario 1 (S1) to scenario 6 (S6). ${\mathbf {G}}$G: additive genomic kernel relationship matrix. ${\mathbf {D}}$D: dominance genomic kernel relationship matrix. $h^2_{AD}$hAD2: broad-sense heritability including additive and dominance variation. Figure S2. Relationship between prediction accuracies (left panel) and connectedness measures (right panel) under an additive and dominance scenario based on the K-means clustering using the multikernel genomic and dominance relationship matrix. The magnitude of the relationship level was steadily increased from scenario 1 (S1) to scenario 6 (S6). ${\mathbf {G}}$G: additive genomic kernel relationship matrix. [file 12711_2018_415_MOESM1_ESM.pdf]

Supplementary Figures

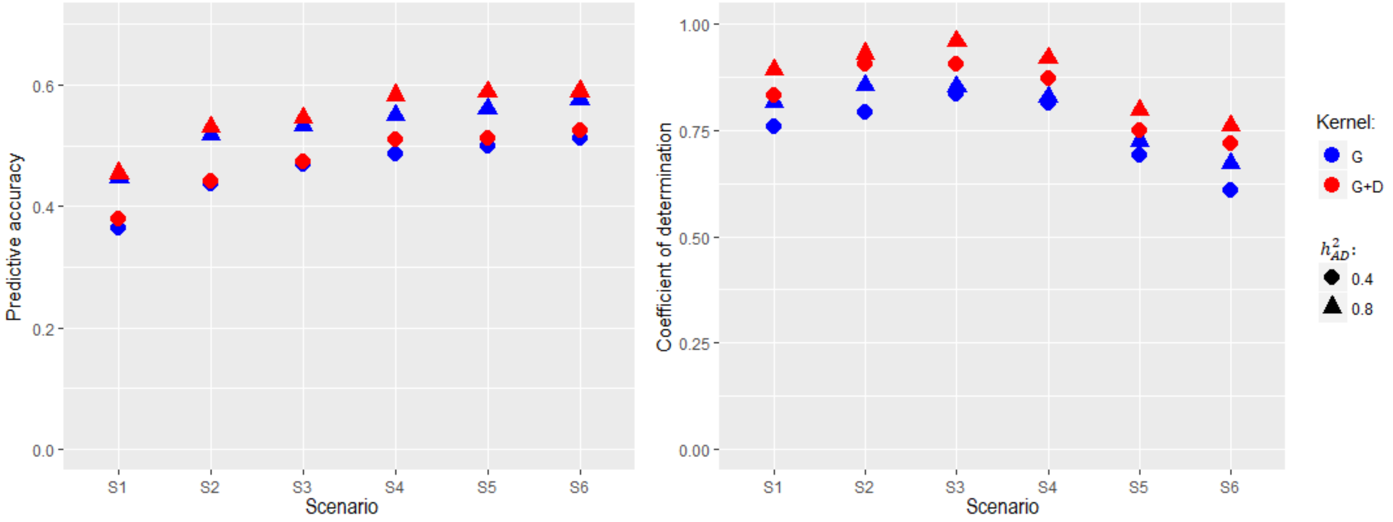

Figure S1: Relationship between prediction accuracies (left panel) and connectedness measures (right panel) under an additive and dominance scenario based on the K-means clustering using the genomic relationship matrix. The magnitude of the relationship level was steadily increased from scenario 1 (S1) to scenario 6 (S6). **G**: additive genomic kernel relationship matrix. **D**: dominance genomic kernel relationship matrix. h^2_{AD}: broad sense heritability including additive and dominance variation.

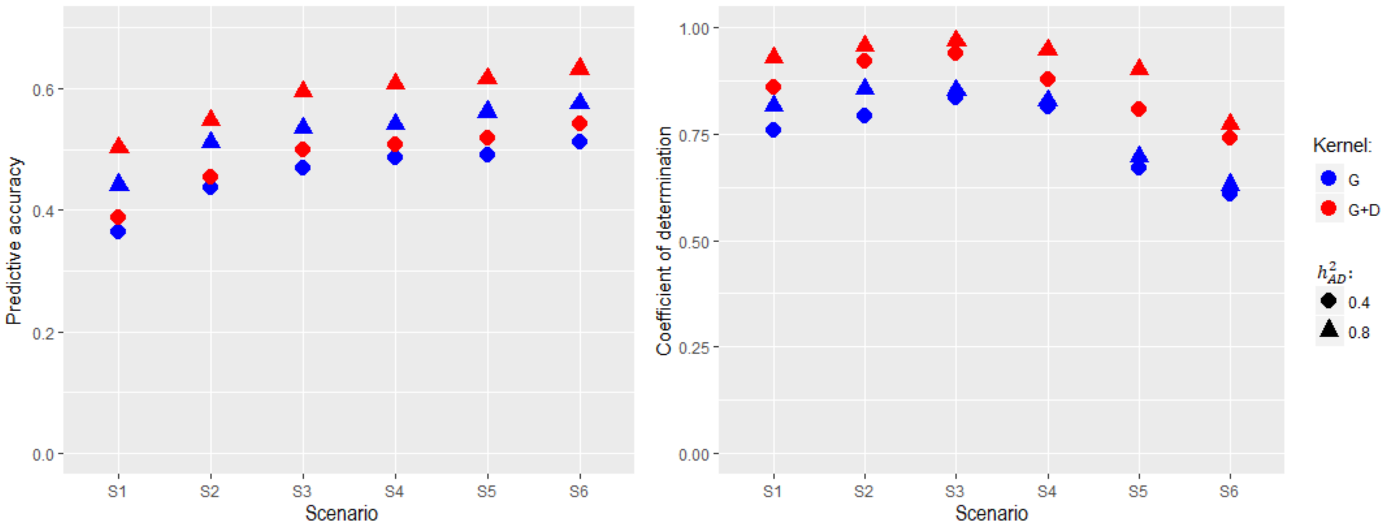

Figure S2: Relationship between prediction accuracies (left panel) and connectedness measures (right panel) under an additive and dominance scenario based on the K-means clustering using the multi-kernel genomic and dominance relationship matrix. The magnitude of the relationship level was steadily increased from scenario 1 (S1) to scenario 6 (S6). **G**: additive genomic kernel relationship matrix. **D**: dominance genomic kernel relationship matrix. h^2_{AD}: broad sense heritability including additive and dominance variation.

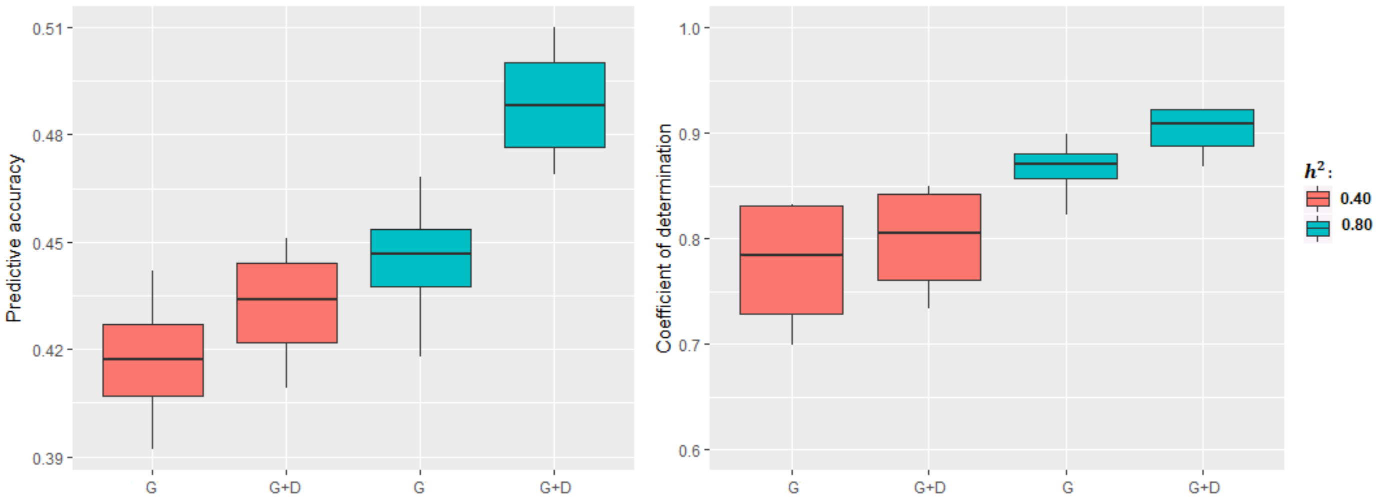

Figure S3: Relationship between prediction accuracies (left panel) and connectedness measures (right panel) under an additive and dominance scenario based on forward validation. **G**: additive genomic kernel relationship matrix. **D**: dominance genomic kernel relationship matrix. h^2: heritability.
